# A review on radiological properties of fused deposition modelling material for three-dimensional printing in proton and light ion beam therapy

**DOI:** 10.1016/j.phro.2026.101042

**Published:** 2026-07-18

**Authors:** Christina Stengl, Christina Mooshammer, Jonas Mahnke, Armin Runz, José Vedelago

**Affiliations:** a Department of Radiation Oncology, Heidelberg University Hospital (UKHD), Im Neuenheimer Feld 400, Heidelberg, 69120, Germany; b Department of Medical Physics in Radiation Oncology, German Cancer Research Center (DKFZ), Im Neuenheimer Feld 280, Heidelberg, 69120, Germany; c Heidelberg Institute for Radiation Oncology (HIRO), National Center for Radiation Research in Oncology (NCRO), Heidelberg, 69120, Germany; d Department for Physics and Astronomy, University of Heidelberg, Im Neuenheimer Feld 226, Heidelberg, 69120, Germany

**Keywords:** 3D printing, Fused deposition modelling (FDM), Stopping power, Electron density, CT number

## Abstract

**Background and Purpose:**

Fused Deposition Modelling (FDM) three-dimensional (3D) printing offers a flexible and economical method for producing radiotherapy phantoms with tailored geometries and material properties. While numerous studies have focused on imaging or photon radiotherapy, research on 3D printing for proton and light ion beam therapy remains limited. Accurate knowledge of the radiological properties of FDM-printed materials is crucial to ensure reliable dose calculation and treatment planning in ion beam therapy.

**Materials and Methods:**

A comprehensive literature review was conducted to identify publications reporting relevant radiological parameters, including mass density, computed tomography (CT) number given in Hounsfield units (HU), electron density, and stopping power, for FDM printing filaments. Based on the collected data, an FDM lookup table was generated, summarising the radiological properties of these materials across different printing settings.

**Results:**

A total of 17 material classes comprising 70 distinct filaments were analysed and indexed in an open-access lookup table. Polylactic acid (PLA) was the most frequently investigated material, reported in over 34 publications. Among the investigated radiological parameters, the CT number showed the greatest variability for a given material. For samples printed at 100% infill, values ranged from -180 HU to 227 HU for PLA. Recommendations for reducing this variability through standardised reporting are provided.

**Conclusion:**

This review provides an overview of FDM 3D printing materials in ion beam therapy. It serves as a practical reference for clinical personnel, medical physicists, and researchers in selecting suitable materials for radiotherapy applications. Moreover, it highlights the need for standardised characterisation methodologies and 3D printing guidelines.

## Highlights

• The literature review is summarised in terms of the relevant radiological properties.
• Lookup table summarising over 70 materials for 3D printing in radiotherapy.
• At least twelve 3D printing settings influence the radiological properties.
• Recommendations for standardised reporting of 3D printed materials.

## Graphical abstract

**Figure:**
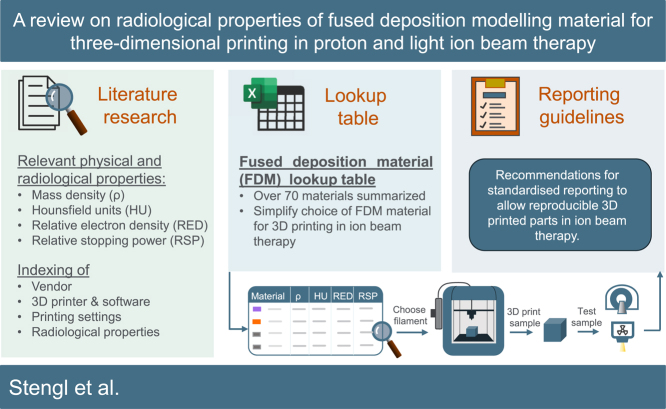

## 1 Introduction

The integration of three-dimensional (3D) printing technology into radiotherapy has significantly advanced the customisation of patient-specific devices, such as boluses, immobilisation tools, and dosimetric phantoms [1], [2], [3]. The widespread adoption of Fused Deposition Modelling (FDM) printing in medical applications is largely driven by its cost-effectiveness [1], [4]. Material costs varied substantially between printing technologies. FDM materials were generally the most economical, with prices starting at approximately 20 EUR per kilogram, whereas PolyJet materials were considerably more expensive, with costs reaching up to 400 EUR per kilogram [5]. In contrast, specialised FDM materials containing functional additives can reach costs of up to approximately 900 EUR per kilogram [6], [7]. Another advantage of FDM is the broad variability in printer models, filament types, and composite materials with different additives. Additional variability arises from the choice of slicing software and the adjustment of its parameters. For the process of FDM 3D printing, digital models are transformed into physical structures layer by layer through the heating and extrusion of various thermoplastic filaments. FDM allows rapid fabrication from a diverse selection of commercially available or custom-modified filaments [8], [9]. Of particular interest is the role of 3D printing in the fabrication of patient-specific medical phantoms used in imaging, dosimetry, and quality assurance. These phantoms play a critical role in validating treatment plans, calibrating imaging systems, and verifying dose distributions, thereby supporting both clinical and research activities [10], [11], [12], [13], [14], [15]. While many existing phantoms are optimised for photon-based treatments, their performance in ion beam therapy can be limited due to the dependence of charged particle interactions on elemental composition [16], [17]. Additionally, an increasing number of phantoms specifically designed for particle therapy, particularly proton therapy, are being developed using 3D printing. These include approaches based on 3D-printed shells filled with water or tissue-equivalent matrices [14], [18], as well as fully solid phantoms employing materials similar to those used in photon therapy [19]. Furthermore, novel material compositions are being explored to better account for particle-specific interactions, such as neutron production within the phantom material [20] or specialised lung tissue to simulate the deterioration of the Bragg peak [21].

For proton and light ion beam therapy, precise characterisation of the radiological properties of FDM-printed materials is essential. Key parameters include computed tomography (CT) number given in Hounsfield units (HU), which reflects the material’s attenuation on CT scans, the relative electron density (RED), influencing photon interactions, and the relative stopping power (RSP), giving ion beam energy loss and range [22], [23], [24]. These parameters are the relevant radiological properties for protons and light ion beams and in the following are referred to radiological properties. To provide further detail on each of these radiological properties, the following sections present a brief overview of the measurement methods, together with relevant references.

CT imaging yields a distribution of X-ray attenuation values referenced to water and expressed in CT number. These CT numbers are directly related to the material’s linear attenuation coefficient, which is governed by its electron density and elemental composition [25], [26], [27]. The CT values vary not only with the material properties but also with the tube voltage, typically expressed in units of kV, used by the CT scanner. Consequently, variability arises not only from the 3D printed part itself but also from the imaging system and its acquisition settings. As the voltage increases, the measured CT number typically decreases [28], [29]. To obtain reproducible CT number measurements, a homogeneous phantom is required around the 3D printed sample to minimise image artefacts and reduce environmental influences while mimicking the attenuation characteristics of the human body. Moreover, the materials must be positioned consistently within the scanner, as variations in placement can also lead to changes in the CT values [25].

For precise CT-based dose calculation in proton and light ion beam therapy, it is necessary to establish a relationship between the photon attenuation coefficients and the ion’s stopping power [30], [31]. These calibration relationships are essential input data for treatment planning systems (TPS), enabling accurate modelling of the beam path and Bragg peak through 3D printed material [25], [32]. RED reflects the electron density of a material relative to water. Since the primary ions mainly lose energy via interactions with electrons, RED is a key parameter in estimating the stopping power of tissues. Typically, a calibration curve is used to convert CT numbers obtained from a specific CT scanner at a given energy into RED values [27]. Two main approaches are commonly employed to establish this calibration curve. The first is the tissue-substitute method, in which a phantom containing tissue-equivalent materials with known physical properties is scanned. The second approach is stoichiometric calibration, which derives the relationship using mathematical models and equations [25], [33], [34]. Single-energy CT enables estimation of RED from CT numbers values. However, the CT number is influenced not only by the electron density of the material but also by its effective atomic number. To improve the accuracy of RED determination, dual-energy CT is frequently used [35], [36]. In addition, consensus guidelines are available for estimating RED from CT number lookup tables in proton therapy [37].

The RSP describes the energy loss of a particle per unit path length in a given material, relative to water. This energy loss can be estimated with the Bethe–Bloch equation and depends on electron density, meaning that any uncertainty in RED propagates directly into an uncertainty in particle range. Since the RSP determines the depth at which the primary beam deposits its maximum dose, a mismatch between the RSP of the 3D printed sample and the RSP assumed in the TPS will result in a Bragg peak displacement. RSP is most commonly measured by placing the 3D printed sample in the particle beam path and quantifying the resulting shift in the percent depth dose (PDD) curve. Typically, the shift in the distal 80% PDD position is divided by the physical thickness of the material to determine the RSP. Measurements can be performed using a single ionisation chamber for depth-dose scanning in a water phantom, or with a dual-ionisation chamber setup, in which one chamber remains stationary while the other moves within a water column. [30], [38], [39], [40].

Although the relationships between CT value, RED and RSP are not strictly linear, they are commonly approximated using piecewise-linear calibration curves composed of two linear segments, one for soft-tissue–like materials and another for bone-equivalent materials [25], [26], [41].

Especially for anthropomorphic phantoms, matching the clinical CT number to RSP relationship can be challenging, as commonly used PLA-based materials often exhibit RSP values that are slightly elevated relative to their corresponding CT numbers in standard clinical calibration curves in the soft-tissue range, while in the higher-density (bone-equivalent) range a tendency towards a decreased RSP compared to the expected values is often observed [24], [40].

Since FDM filaments are not standardised, differences in the composition of the polymer from different vendors and variations in 3D printing parameters, including infill density, infill pattern, and layer thickness, can substantially alter their physical and radiological properties, making systematic characterisation essential [42], [43], [44]. Especially in proton and light ion beam therapy, this becomes important, as variations in these parameters directly affect dose calculation accuracy in TPS, where the precision of the ion range is critical for properly covering the target. Studies have shown that some conventional materials introduce substantial errors in parameters such as CT number and RSP, leading to clinically significant range uncertainties [40], [45].

To date, various review articles have been published on 3D printing techniques [46], [47] and on 3D printing materials themselves, including their mechanical properties, but without addressing their radiological characteristics, especially for ions [4], [48]. Other reviews have focused on medical applications of 3D printing technology, such as bolus fabrication [49], [50], on the use of various medical imaging modalities for assessing 3D-printed objects, including CT, Magnetic Resonance Imaging (MRI), and Positron Emission Tomography (PET) [22], and on 3D-printed medical phantoms for imaging and dosimetry [3]. However, no comprehensive review has yet been conducted on FDM materials specifically for phantom fabrication in ion beam therapy. Existing data on different materials and printing conditions remain fragmented and inconsistent, posing challenges for the clinical adoption of FDM-printed devices.

This review aimed to summarise current data on a wide range of FDM-printed materials that can be used in proton and light ion beam therapy, covering vendor information, 3D printing parameters, including bed and nozzle temperature, and radiological properties, including CT number, relative electron density, and relative stopping power. To streamline the selection of suitable materials for phantom 3D printing, a FDM lookup table is provided as a reference for clinical personnel and researchers to support the design of phantoms, optimising the application of FDM 3D printing in this field. Moreover, this review aimed to give recommendations on how to and what to report when using FDM 3D printing phantoms for proton and light ion beam radiotherapy.

## 2 Materials and methods

### 2.1 Literature search

For the literature search, the databases PubMed and Scopus were used. The search strategy included the keywords “3D printing”, “Fused deposition modelling”, “radiotherapy”, “proton therapy”, “ion beam therapy”, “electron density”, “stopping power”, “Hounsfield”, “CT number” in different combinations and with abbreviations (HU, RED, RSP) from 2015 to 2025. Since this review focused on FDM 3D printing, other additive manufacturing techniques like Stereolithography and PolyJet printing were not considered [11]. From the initial search, studies that did not report the infill of the 3D-printed structures were not considered. Additionally, studies involving in-house-made filaments were excluded from consideration [51]. Finally, a total of 54 publications [9], [23], [24], [42], [43], [44], [52], [53], [54], [55], [56], [57], [58], [59], [60], [61], [62], [63], [64], [65], [66], [67], [68], [69], [70], [71], [72], [73], [74], [75], [76], [77], [78], [79], [80], [81], [82], [83], [84], [85], [86], [87], [88], [89], [90], [91], [92], [93], [94], [95], [96], [97], [98], [99], [100] resulted from this search and were included in this review.

### 2.2 FDM lookup table and FDM material classes

For creating the FDM lookup table, information on vendor, chemical composition, mass density, 3D printer model, extrusion factor/flow rate, extrusion and bed temperatures were considered, along with the radiological parameters such as printed mass density, CT numbers at 80 kV, 100 kV, 120 kV and 140 kV, RED and RSP, from the 54 selected publications [9], [23], [24], [42], [43], [44], [52], [53], [54], [55], [56], [57], [58], [59], [60], [61], [62], [63], [64], [65], [66], [67], [68], [69], [70], [71], [72], [73], [74], [75], [76], [77], [78], [79], [80], [81], [82], [83], [84], [85], [86], [87], [88], [89], [90], [91], [92], [93], [94], [95], [96], [97], [98], [99], [100].

In this review, a total of 17 material classes were summarised (Table 1), including 70 different FDM materials published in a dataset [101]. Statistical data on the number of studies using each material class were collected, as well as the reported ranges of extrusion and bed temperatures used during printing. Comparisons of mass density between the raw filament material and 3D-printed parts were also considered. The uncertainties given are the standard deviation of the mean as reported in each study.

A more detailed analysis was done for PLA and ABS, integrating data from multiple studies. This included assessments of the influence of filament colour and polymer additives on the measured radiological quantities. Finally, based on the variety of data in the analysed publications, this review proposed recommendations for reporting 3D-printing parameters for applications in proton and light ion beam therapy.

**Table 1:** Typically used raw FDM 3D printing filaments, along with their abbreviations and their chemical formulas. *CPE is used as an abbreviation for chlorinated Polyethylene (PE) and for chlorinated Polyethylene terephthalate (PET). TPC, TPE and TPU do not have a specific chemical formula.

Abbreviation	Full name	Chemical formula	
ABS	Acrylonitrile butadiene styrene	(C_15_H_17_N)${}_{n}$	[24]
ASA	Acrylonitrile styrene acrylate	(C_18_H_23_NO${}_{2}$)${}_{n}$	[81]
BVOH	Butenediol vinyl alcohol copolymer	(C${}_{4}$H${}_{8}$O${}_{2}$⋅C${}_{2}$H${}_{4}$O)${}_{n}$	[102]
CPE	Chlorinated polyethylene*	(C_4_H${}_{7}$Cl)	[103]
HIPS	High Impact polystyrene	(C${}_{8}$H${}_{8}$)${}_{n}$	[52]
–	Nylon	(C_12_H_22_N${}_{2}$O${}_{2}$)${}_{n}$	[52]
PC	Polycarbonate	(C_16_H_14_O${}_{3}$)${}_{n}$	[104]
PET	Polyethylene terephthalate	(C_10_H${}_{8}$O${}_{4}$)${}_{n}$	[104]
PETG/PEDG	Polyethylene terephthalate glycol	(C_26_H_26_O${}_{8}$)	[24]
PLA	Polylactic acid	(C${}_{3}$H${}_{4}$O${}_{2}$)${}_{n}$	[52]
PMMA	Polymethyl methacrylate	(C${}_{5}$H${}_{8}$O${}_{2}$)${}_{n}$	[24], [52]
PP	Polypropylene	(C${}_{3}$H${}_{6}$)${}_{n}$	[104]
PVA	Polyvinyl alcohol	(C${}_{2}$H${}_{4}$O)${}_{n}$	[52]
TPC	Thermoplastic copolyester	–	
TPE	Thermoplastic elastomer	–	
TPU	Thermoplastic polyurethane	–	
Vinyl (PVC)	Polyvinyl chloride	(C${}_{2}$H${}_{3}$Cl)${}_{n}$	[105]

## 3 Results

### 3.1 FDM lookup table and FDM material classes

A lookup table for the different FDM material classes is provided as a dataset [101]. This table allows for searching the printing parameters used to gain a certain radiological parameter and enables simplifying material decisions upon phantom construction. It is intended to serve as a preliminary guide to reduce the time and effort involved in selecting suitable materials for specific radiotherapy applications. While information on CT number is widely available, other radiological parameters such as RED and RSP relevant for ion therapy are rather sparse and therefore not all of the FDM lookup table was filled.

**Fig. 1:**
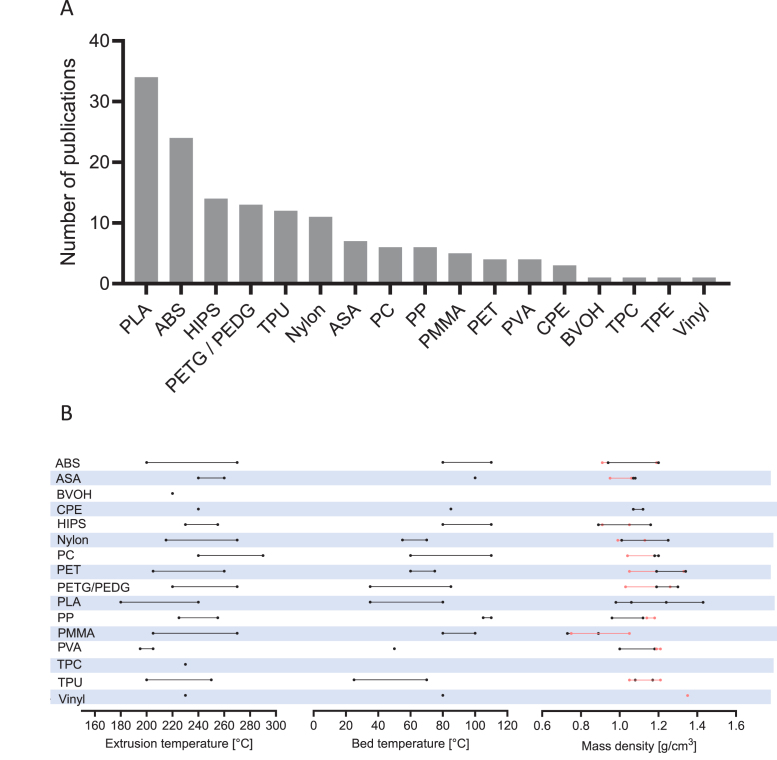
Number of publications used for this review mentioning the given material class on the x-axis (A). Range of extrusion temperature, bed temperature, nominal (black) and printed mass density (orange) for each material class (B). Temperature and density were not reported for any additives.

FDM materials comprise a wide range of filaments with varying chemical structures. Regarding chemical composition (Table 1), it should be noted that the values represent the nominal base formulation of the raw FDM material and do not account for additives or fillers that may be present in commercial filaments. The most commonly used FDM filament is polylactic acid (PLA), widely favoured for its ease of printing and stability [106]. PLA was mentioned in over 78% of the studies summarised in the FDM lookup table (Fig. 1 A). Acrylonitrile butadiene styrene (ABS) ranks as the second most frequently used filament in medical radiotherapy applications, appearing in approximately 59% of the previous publications. Compared to PLA, ABS provides greater mechanical strength and heat resistance but presents challenges such as warping during printing [48], [107].

Following ABS, polyethylene terephthalate glycol (PETG/PEDG) accounts for 29% of the reported usage. PETG is valued for its toughness and flexibility. However, its tendency to absorb moisture and susceptibility to surface scratching can be disadvantages [48], [108]. Other materials such as nylon and thermoplastic polyurethane (TPU) offer enhanced flexibility and elasticity, making them particularly suitable for fabricating deformable parts or simulating mechanically compliant soft tissues [109], [110]. Less commonly used materials include thermoplastic copolyester (TPC), thermoplastic elastomer (TPE) and vinyl.

Extrusion temperatures varied considerably across studies. For example, while manufacturers typically recommend an extrusion temperature from 230 °C to 255 °C for ABS, reported studies have used temperatures ranging from 200 °C to 270 °C [87]. A similar trend was observed for PLA. The manufacturer’s advised range is 190 °C to 230 °C [93], yet studies reported using 180 °C to 260 °C (Fig. 1 B). Moreover, formulation-specific variants within a material class can further widen the usable temperature window, underscoring the importance of accurately reporting printing conditions.

Bed temperature is another critical parameter, as incorrect settings can lead to poor adhesion or overheating. Bed temperature requirements also influence the feasibility of multi-material printing. For instance, ABS, HIPS, and PP have similar recommended bed temperatures (Fig. 1 B), enabling them to be combined more easily. In contrast, PLA has a non-overlapping bed temperature range, complicating its use in multi-material prints.

Besides the material classes of the filament, differences between printer models, nozzle diameter and nozzle material can influence the measurable outcome of 3D printed materials, even when the same filament and slicing parameters are used [54].

### 3.2 Radiological properties of FDM materials

Table 2 provides an overview of the different material classes discussed above, including the reported ranges of radiological properties, CT number, RED, and RSP, and summarises the single values provided in the FDM lookup table, where individual variations are reported. The references for each parameter and material class are given in Table S1. The CT number exhibits wide variability, often spanning ranges of 200 to 400 HU. This variability can largely be attributed to differences in imaging systems, printer models, and printing parameters across studies. In contrast, the RED values show less variation. However, the available data are limited, with detailed information reported primarily for the most frequently used materials PLA, ABS and PETG. Information on RSP is even more scarce.

**Fig. 2:**
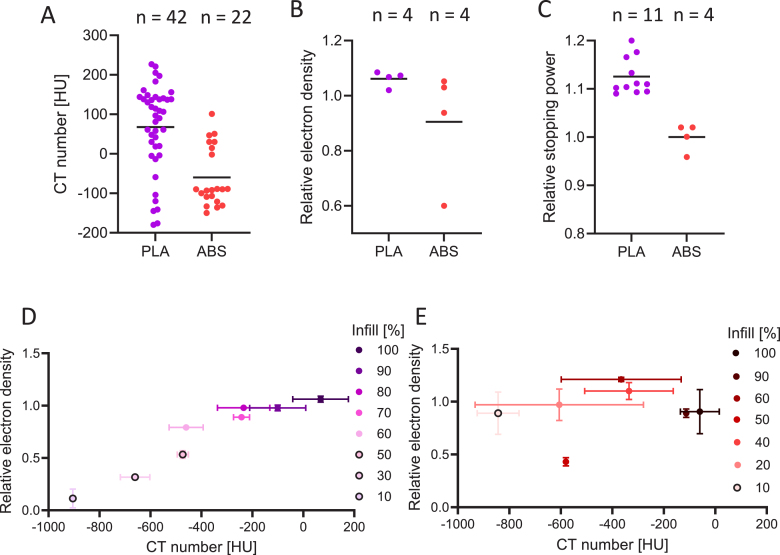
CT number for PLA and ABS (A), RED for PLA and ABS (B) and RSP for PLA and ABS (C) are shown. The number of reported values over the analysed studies is given by “n”. For (A) to (C), each dot represents a reported value and the median value is represented with a horizontal black line. RED vs. CT number for different percentages of infill for PLA (D) and RED vs CT number for ABS (E). For (E) and (D) the error bars show the standard deviation over all reported values. References for each value is given in Table S2-S5.

The variability in CT number values, RED and RSP for PLA and ABS is depicted in greater detail in Fig. 2 A-C. The references are given in Table S2-S5. The CT numbers for PLA ranged from -180 HU to 226 HU, with a mean of 68 HU and a standard deviation of 109 HU. Instead, the CT numbers for ABS were confined to a narrower interval from -150 HU to 101 HU, with a mean of -60 HU and a standard deviation of 76 HU. In comparison, the reported RSP values demonstrated lower variability for PLA and ABS. PLA typically demonstrated radiological properties similar to those of soft tissue with CT number in the range of 20 HU to 100 HU [111] and RSP of 1.049 to 1.058 [112], making it suitable for the fabrication of patient-specific boluses and phantoms [62], [113]. ABS, in contrast can be used to imitate fat tissue with CT numbers of -100 HU to -80 HU and an RSP of 0.957 [52], [111], [112].

An intended reduction in CT number can be achieved by decreasing the infill of the 3D-printed structure, thereby decreasing the amount of material and increasing the proportion of air, which in turn reduces both CT number and RED (Fig. 2 D,E) [42], [43], [44], [53], [55], [58], [62], [63], [68], [77], [114]. Through such controlled porosity or by selecting filaments with intrinsically low mass density, materials can be engineered to approximate lung tissue, which typically exhibits CT values of –950 to –550 HU [111] and RSP values between 0.192 and 0.513 [112].

**Table 2:** Radiological properties of common FDM 3D printing materials, including CT numbers measured with 120 kV, RED, and RSP. Values for additives added to the different material classes were not included in this overview. The references for CT number, RED and RSP are given in Table S1.

Material	CT number [HU]	RED	RSP
ABS	−150 to 101	0.6 to 1.09	0.96 to 1.02
ASA	−184 to 155	0.83 to 0.97	
BVOH	1		
CPE	47 to 160	1.08	
HIPS	−166 to −42	0.92	0.91
Nylon	−117 to 94	1.04 to 1.08	
PC	−15 to 140		
PET	31 to 274		
PETG	−63 to 250	0.98 to 1.02	1.02 to 1.18
PLA	−180 to 299	1.02 to 1.09	1.1 to 1.2
PMMA	−136 to 56	1.01 to 1.03	1.08
PP	−435 to −121	0.72	
PVA	−52 to 229		
TPC	−70		
TPE	−235		
TPU	−216 to 136	0.98	
Vinyl	996		

**Fig. 3:**
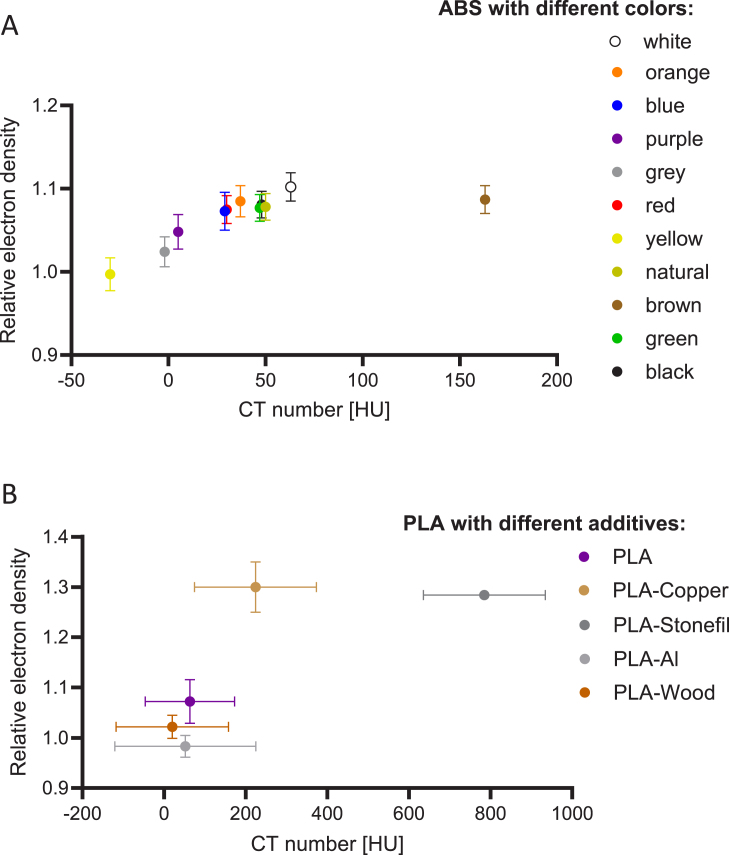
Influence of the ABS filament colours in the relationship between RED and CT number (A) [42], [79]. Each point represents a filament colour with the colour of the filament itself. Influence of additives on the relationship between RED and CT number for PLA, PLA with copper (PLA-Cu), PLA with stone (PLA-Stonefil), PLA with aluminium, and PLA with wood (B). The references used for the values are listed in Table S6.

It is possible to change the CT number while keeping the RED similar by changing the colours from the same material class and supplier [42]. The small amount of additives used to achieve different colours can alter the CT number, but has only a minor effect on RED (Fig. 3 A). This should be taken into account when printing human-like tissue, as not every colour aligns with the desired CT-to-RED relationship.

Although these additives expand material diversity, it should be noted that silicates, wood, and metal fillers are compositionally further from human tissue than standard FDM thermoplastics. Consequently, materials with higher effective atomic numbers tend to show larger discrepancies between the RSP values predicted by clinical lookup tables and the experimentally determined RSP [44].

## 4 Discussion

This review summarised the 3D printing settings and resulting radiological properties of FDM materials used in the fabrication of phantoms for proton and light ion beam therapy. Among the materials reviewed, PLA was the most frequently investigated for radiotherapy applications. However, considerable variability in CT numbers was observed for PLA at equivalent infill densities across different studies, highlighting challenges in achieving consistent radiological properties. Furthermore, the incorporation of additives substantially altered both CT number and RED, demonstrating the potential to tailor material properties for specific phantom applications while emphasising the need for careful material characterisation.

The FDM lookup table consolidates information on 3D printers, slicer software, and printing settings alongside corresponding radiological parameters, providing a first step towards the clinical integration of FDM-based phantoms in proton and light ion beam therapy [101]. It allows readers to identify previously investigated FDM materials that best match their specific needs, while also drawing attention to the considerable variability currently reported in the literature and pointing to existing datasets that may serve as a basis for future standardisation. In the longer term, this table could support the development of a centralised database or an online tool capable of predicting key radiological properties from defined printing parameters.

In this context, the recently published report provides comprehensive recommendations for quality assurance and standardisation throughout the medical 3D printing workflow [115]. The present review complements these guidelines by providing a comprehensive summary of the radiological properties of over 70 materials for 3D printing, along with a practical lookup table [101]. Moreover, it gives recommendations for standardised reporting for 3D printing in proton and light ion beam therapy (Table 3).

While there is a consensus that 3D printing technology holds significant potential for clinical applications in the fabrication of phantoms for medical physics [22], [116], methodological inconsistencies and gaps in reporting practices currently limit reproducibility and clinical applicability. Details such as filament composition, printer specifications, and printing parameters are frequently omitted. The lack of such data limits reproducibility and prevents meaningful comparison between studies.

**Table 3:** Recommendations for standardised reporting of parameters for FDM 3D printing in radiological applications during proton and light ion beam therapy.

Category	What to report	Rationale
Filament composition	Base polymer and additives/colour; exact filament name; vendor and purchase date; chemical composition (if available)	Composition varies between manufacturers and over time; affects mass density, CT number, RED, and RSP; essential for reproducibility.
Material naming	Unambiguous material definitions	Prevents confusion caused by identical abbreviations assigned to different chemical structures.
3D printer hardware	3D printer model and manufacturer; nozzle diameter/material, number of toolheads	Different printers can induce measurable variability in radiological properties even with identical filament and settings.
3D printing parameters	Extrusion and bed temperatures; print speed; extrusion factor; infill density and pattern; layer height; cooling setting; enclosure; slicer software and version number	Printing conditions significantly influence microstructure, foaming behaviour, warping, adhesion, and consequently radiological properties.
Sample geometry	Dimensions; perimeter and thickness; orientation	Geometry affects bulk density, CT number accuracy, edge effects, and the occurrence of imaging artefacts.
CT imaging conditions	CT scanner model; acquisition protocol, tube voltage, tube current, reconstruction kernel, slice thickness; phantom setup; sample positioning	CT numbers are energy-dependent and sensitive to scatter and beam hardening; essential for comparability across studies.
Radiological measurements	CT number, RED, and RSP values; measurement method and analysis software; uncertainties	RSP is critical for proton and ion therapy planning; uncertainties must be reported for reliable dosimetric use.
Reproducibility and stability	Number of replicates; intra-/inter-sample variability; vendor-to-vendor differences; long-term stability	Required to assess the reliability, reproducibility, and robustness of reported data.
Multi-material printing	Interface quality; possible voids; adapted cooling or bed temperature settings	Material interfaces can produce density discontinuities and impact radiological behaviour.
Uncertainties	Statistical and experimental uncertainties, including standard deviation within the region of interest, reproducibility between repeated prints, and uncertainties related to the measurement setup, imaging, and analysis	To improve reproducibility, comparability, and interpretation across studies

One key point is the specification of the filament composition, including both the base polymer and any additives. As nominally identical FDM materials can be purchased from multiple vendors, it is essential to report the exact filament name, manufacturer, date of purchase and colour of the filament [42]. Vendor portfolios change frequently, and such fluctuations can compromise reproducibility. Moreover, since FDM materials are generally not protected by proprietary formulations, their composition and mass density may vary slightly between suppliers. These differences can influence critical radiological parameters such as mass density, CT number, and RED [42]. Therefore, switching vendors without re-evaluating the material is not recommended. Furthermore, when feasible, assessing the chemical composition can be advantageous, for example, when implementing the phantom in Monte Carlo simulations [52]. Another important aspect is the careful and unambiguous definition of material names, as the same abbreviation has been used in past studies to refer to different chemical structures. For example, the abbreviation CPE has been used for two different chemical compositions, chlorinated PET [55] and chlorinated PE [59], [86].

Variability between printers can lead to measurable differences in radiological properties, underlining the importance of reporting hardware specifications [54]. The FDM lookup table [101] highlights the great variety of 3D printer models used across all studies. In addition to the hardware, the software can also play an important role, particularly the choice of slicer software and its version number [117].

3D printing parameters significantly influence the physical and radiological properties of the printed part or phantom. Specifically, variations in extrusion and bed temperatures can modify the material’s structure and density. For instance, in lightweight PLA, a PLA variant engineered for low-density applications, differences in extrusion temperature alter the extent of material foaming, which subsequently affects key radiological parameters [64], [118]. Excessive bed temperatures can induce thermal warping, leading to deviations in the intended geometry, while suboptimal bed temperatures may result in insufficient adhesion to the build platform, compromising dimensional accuracy and structural integrity [119]. This is also linked to the temperature in the printing room, which should be reported. Especially ABS is recommended to print with an enclosure to keep the part at a certain temperature to make adhesion to the printing bed and warping less likely. In addition to temperature, the extrusion factor has a strong influence on the radiological properties as it changes the infill of the 3D printed part [120]. During the 3D printing process, air can be trapped within the printed structure, leading to undesired effects on the radiological properties relevant for ion beam therapy. Air pockets that are not visible in the CT can change the effective density and therefore affect the ion beam range and dose distribution compared to the treatment planning CT. In addition, anisotropic printing, depending on the build direction, can lead to uneven void formation and further increase these differences [74], [121].

For the radiological parameters, however, not only are the material and 3D printing parameters of relevance, but also the techniques used to analyse these parameters. Among these quantities, the CT number is particularly sensitive to imaging parameters, most notably the tube voltage used during acquisition. Consequently, the CT numbers of printed materials exhibit a clear energy dependence. Recent studies characterised materials across multiple tube voltages and have shown that CT number can vary considerably with beam energy [42], [73]. Another point to be considered is that many studies acquire CT data from isolated printed samples positioned directly on the scanner couch without surrounding phantom materials. As reported by previous investigations, this approach can lead to inaccuracies due to altered scatter conditions and beam hardening corrections [77]. The geometry of the 3D-printed sample can also influence the measured CT numbers. If samples are too small, edge effects may interfere with the analysis and lead to inaccurate CT number assessment. These issues can be mitigated by following established recommendations for CT-based prediction of RSP in proton therapy, which have already been published [37]. Moreover, when the infill density is below 100%, the slicer software typically adds one or more outer shell perimeters, which can alter the effective bulk density of the 3D-printed part [122]. Consequently, it is essential to report the sample geometry in detail, including dimensions and perimeter settings, to ensure accurate interpretation and reproducibility. The layer-by-layer fabrication principle inherent to FDM introduces additional geometric limitations. The spatial resolution of FDM printing typically ranges between 100μm and 500μm, which is lower than that of other 3D printing technologies [123], [124]. This limitation can produce stair-step effects, which may appear as artefacts on CT images. Such artefacts can compromise the accuracy of treatment planning and dose calculations [125].

Due to variations in 3D printing settings and imaging setup, it is strongly advisable to perform validation measurements of predefined materials under local conditions before clinical use. Therefore, a quality assurance guideline already exists, providing recommendations for equipment, software, and workflow design [126]. Additionally, depending on the intended application, long-term stability testing of the material may be relevant. While the chemical composition is generally expected to remain stable under irradiation, the mechanical properties of the material may change with accumulated dose [127], [128], [129]. Moreover, comprehensive reporting of reproducibility, including the number of replicates and any observed intra- or inter-sample variability, is essential for assessing the reliability and robustness of the results.

A substantial number of studies have investigated CT numbers. However, RED [23], [24], [42], [43], [44], [55], [62], [63], [72], [74] and RSP, as needed for proton and light ion beam therapy, are less frequently addressed [24], [60], [61], [71], [72], [74], [75], [76], [78]. 3D printing is widely used in imaging and photon beam therapy, yet its application in proton and light ion beam therapy remains comparatively limited. In these modalities, reliance on CT number alone is insufficient, as RSP data are crucial for treatment planning and dose calculation [130]. Consequently, FDM-printed materials require more detailed characterisation with respect to their RSP. With the increasing number of proton and, more recently, light ion therapy centres, the demand for suitable phantoms is expected to grow significantly, further underscoring the need for well-characterised 3D-printed materials [114], [131].

To advance the development of anthropomorphic phantoms for proton and light ion beam applications, recent progress in multi-material 3D printing is highly promising. However, it also introduces additional methodological complexity [132]. The interface between different materials within a single printed object can contain voids or weak adhesion zones that are rarely characterised but could influence both mechanical integrity and radiological properties [133]. In proton and ion beam therapy applications, the presence of air inclusions within the printed material can alter RSP and, therefore, requires systematic investigation before clinical use. Adjustments such as modifying the cooling fan speed or fine-tuning the bed temperature towards the other material’s range may be necessary to achieve optimal results. Another approach for improving phantom or bolus fabrication is the use of in-house produced FDM filaments, as explored in a few studies [8], [54], [134], [135], [136]. Such approaches may enable a wider range of achievable physical and radiological properties, while also offering greater control over the filament composition [9]. However, this advantage is only valid if the composition of the raw materials is precisely known and consistently reproducible. In addition, in-house filament production introduces further challenges, including increased time requirements, reproducibility and quality-control considerations, as well as the need for specialised equipment and trained personnel. From a reporting perspective, also more detail must be provided, including the sources of the base polymer and the additives, exact additive composition, mixing and grinding procedures including temperature and speed as well as the equipment used, and storage conditions.

Based on the FDM lookup table, the radiological behaviour of FDM-printed objects is not determined solely by the selected material but is also substantially influenced by printing parameters such as infill density and infill pattern, as well as by the specific techniques used to assess radiological properties, including the chosen CT tube voltage [101]. Consequently, understanding the interplay between material composition, printing conditions, and analytical methods is essential for optimising the use of 3D-printed anthropomorphic phantoms in clinical radiotherapy. For clinical implementation of FDM-based phantoms, future research should prioritise standardisation of both 3D printing and analysis protocols used to characterise them. Optimisation of 3D printing parameters should aim to balance anatomical accuracy, mechanical stability, and radiological tissue equivalence. To address this, we recommend standardised reporting practices. Table 3 provides a summary of the minimum parameters that should be documented to enable reproducibility, facilitate comparisons across studies, and support the clinical translation of FDM printing technologies. Furthermore, Table S7 gives additional parameters and examples on how and what to report.

In conclusion, FDM 3D printing is currently used with limited specifications in proton and light ion therapy for the fabrication of customised phantoms. The radiological properties of FDM-printed materials, such as CT number, relative electron density, and relative stopping power, are strongly influenced by filament composition, 3D printing parameters and analysis methods. The FDM lookup table compiled during the review provides an initial reference for understanding the impact of these parameters and the range of values achieved so far. Furthermore, the proposed recommendations for reporting 3D printing data serve as a foundation for promoting the reproducible and effective use of 3D printing in research and clinical applications.

## CRediT authorship contribution statement

**Christina Stengl:** Writing – review & editing, Writing – original draft, Visualization, Validation, Methodology, Investigation, Data curation, Conceptualization. **Christina Mooshammer:** Writing – review & editing, Validation, Investigation, Formal analysis. **Jonas Mahnke:** Writing – review & editing, Validation, Investigation, Formal analysis. **Armin Runz:** Writing – review & editing. **José Vedelago:** Writing – review & editing, Writing – original draft, Supervision, Project administration, Funding acquisition, Conceptualization.

## Declaration of competing interest

The authors declare that they have no known competing financial interests or personal relationships that could have appeared to influence the work reported in this paper.

The author José Vedelago is an Associate Editor for this journal and was not involved in the editorial review or the decision to publish this article.

## Appendix A Supplementary data

MMC S1
