# Supplementary material for: A review on radiological properties of fused deposition modelling material for three-dimensional printing in proton and light ion beam therapy
Source: Phys Imaging Radiat Oncol. 2026 Jul 18;40:101042. doi: 10.1016/j.phro.2026.101042 (PMC13416827; doi:10.1016/j.phro.2026.101042)
Supplement: MMC S1 [file mmc1.pdf]

Supplementary material

A review on radiological properties of fused deposition modelling material for three-dimensional printing in proton and light ion beam therapy

Christina Stengl, Christina Mooshammer, Jonas Mahnke, Armin Runz, José Vedelago

Table S1: Overview of reported CT number, relative electron density (RED), and relative stopping power (RSP) measurements for different FDM materials. Literature according to Table 2.

Material	CT number	RED	RSP
ABS	[9], [24], [42], [43], [44], [53], [55], [56], [59], [68], [73], [77], [80], [84], [86]	[24], [42], [55], [74]	[24], [74], [76]
ASA	[9], [42], [55], [69], [73], [86]	[42]	–
BVOH	[9]	–	–
CPE	[55], [59], [86]	[55]	–
HIPS	[9], [24], [53], [68], [73], [77], [79], [88], [83], [86]	[24]	[24], [61]
Nylon	[59], [69], [73], [77], [79], [86]	[55]	[61]
PC	[73], [86]	–	–
PET	[73], [86]	–	–
PETG	[24], [53], [55], [59], [69], [70]	[24], [55]	[24], [76]
PLA	[9], [24], [53], [54], [55], [56], [57], [58], [59], [60], [62], [63], [65], [67], [68], [69], [70], [71], [72], [73], [77], [80], [86]	[55], [63], [72]	[60], [61], [71], [72], [75], [76]
PMMA	[24], [42], [54], [86]	[24], [42]	[24]
PP	[9], [55], [57], [59], [73]	[55]	–
PVA	[53], [79], [86]	–	–
TPC	[9]	–	–
TPE	[53]	–	–
TPU	[9], [42], [53], [54], [73], [79], [80], [86]	[42]	–
Vinyl	[73]	–	–

Table S2: Reported CT numbers for PLA materials with literature. Uncertainties, if available, are given in round brackets. Values according to Figure 2A.

CT number [HU]	Reference
138(1)	[9]
58(17), 48(14)	[24]
-180(18), -176(14), -145(28), -141(9), -104(17)	[42]
30(13)	[53]
135.9, 19.6	[54]
18.39(8.07)	[55]
42(8)	[56]
148.4(2.7)	[57]
183(8)	[58]
114.58(11.62)	[59]
161(5)	[60]
-120(31)	[62]
138(12)	[63]
140.6(11.5), 137.8(14.3), 60.9(25.3), 118.4(41.3), 221.0(12.5), -5.1(58.9), -4.0(52.0)	[65]
144	[67]
61	[68]
226.78(14)	[69]
90	[70]
156(40), 144(82), 137(65)	[71]
106.5(15.2), 130.1(10.1)	[72]
205.0, 197.2	[73]
-13.6(132.7)	[77]
97	[80]
-59(19), 81(34), 109(28)	[86]

Table S3: Reported CT numbers for ABS materials. Uncertainties, if available, are given in brackets. Values according to Figure 2A.

CT number [HU] (uncertainty in brackets)	Reference
-89(2)	[9]
-90(12), -107 (10)	[24]
14.6(0.8)	[42]
-133(12)	[53]
30.11(3.05)	[55]
-2(6)	[56]
50.59(9.18)	[59]
30.4	[73]
-150.0(11.1)	[77]
-93	[80]
101(37), -89(25), -136(35), -100(37), -121(41), -109(55), -131(41), -92(48), 47(20), -90(25)	[86]

Table S4: Reported RED and RSP values with literature for PLA and ABS materials according to Table 2B and 2C.

**RED – PLA**	**Reference**
1.02	[55]
1.085	[63]
1.068, 1.073	[72]
**RED – ABS**	**Reference**
0.938(0.019)	[24]
1.03	[55]
1.052(0.001)	[42]
0.6	[74]
**RSP – PLA**	**Reference**
1.111(0.002)	[60]
1.09(0.01)	[61]
1.1038(0.0209), 1.1098(0.0209), 1.0945(0.0379)	[71]
1.093(0.007), 1.102(0.004)	[72]
1.20(0.05)	[75]
1.133(0.970), 1.166(0.962), 1.176(0.958)	[76]
**RSP – ABS**	**Reference**
0.959(0.002)	[24]
0.61	[74]
1.001(0.997), 1.020(1.007), 1.020(1.002)	[76]

Table S5: Reported CT number and RED values for ABS and PLA at different infill densities with corresponding literature. Uncertainties are given in brackets (). ";" refers to a new literature source while those separated by "," are the same source. Values according to Figure 2D, E.

Infill [%]	CT number [HU]	Literature	RED	Literature
**ABS**				
90	−113(3)	[44]	0.89(0.04)	[44]
60	−461(11); −100; −535(12)	[53], [43], [68]	1.21(0.02)	[43]
50	−580(1)	[44]	0.43(0.04)	[44]
40	−334(172)	[43]	1.10(0.08)	[43]
20	−606(327)	[43]	0.97(0.15)	[43]
10	−786(425); −901(1)	[43], [44]	0.89(0.20)	[43]
**PLA**				
90	−262(38), −277(37), −163(11), −222(8), −180(31); 8(4); −53.94(26.55); −60(8.3); −21(19); −30(55); −39(17); 79; −84.2(152.8)	[42], [44], [55], [56], [58], [62], [63], [70], [77]	0.96; 1.01(0.03); 0.963(0.017)	[55], [44], [63]
80	−198.58(27.45); −364(47), −396(57), −268(14), −321(10), −282(26); −181(10), −190(8), −304(12), −257(10), −130(23), −58(19); −95(40)	[42], [53], [55], [62]	0.979(0.005)	[62]
70	−234(5), −288.24(22.15), −228(75), −219(42)	[44], [55], [58], [62]	0.889(0.005)	[62]
60	−530(95), −542(101), −473(9), −499(51), −492(14); −370(7); −404.01(24.68); −360(70); −530(25); −393.7(142.5)	[42], [53], [55], [62], [68], [77]	0.792(0.007)	[62]
50	−466(8); −501.76(22.13); −490(53); −446(187); −453(84); −482(17)	[44], [55], [56], [58], [62], [63]	0.47; 0.54(0.05); 0.527(0.017)	[55], [44], [63]
30	−688(3); −699.49(19.82); −661(256); −693(17); −560.3(373.7)	[44], [55], [58], [63], [77]	0.317(0.017)	[63]
10	−916(1); −901.07(74.61); −898(90); −904	[44], [55], [63], [68]	0.113(0.090)	[63]

Table S6: Reported CT number and RED values for PLA-based composite filaments with additives with corresponding literature according to Figure 3B.

Additive	CT number [HU]	Reference	RED	Reference
PLA-Copper	359(21); 330(19); 119.7	[42], [53], [54]	1.086(0.025)	[42]
PLA-Stonefil	563(11); 735(28), 836(28); 460(24); 968(38); 1063.1; 823(39)	[9], [24], [42], [66], [73], [78]	1.284(0.007); 1.208(0.027)	[24], [42]
PLA-Al	−21(18); −72(15); 249.6	[42], [53], [73]	0.983(0.022)	[42]
PLA-Wood	−31(1), −124(12); 33(17); 203.0	[9], [42], [73]	1.022(0.023)	[42]

Table S7: Recommendations with examples and important questions to ask.

**Category**	**Examples and guiding questions**
Filament composition	Report the nominal base polymer, vendor, colour, and possible additives. Example: Easy PLA, Fiberlogy, transparent, batch date 07.05.2026. If available, include vendor specifications or safety data sheets.
Material naming	Use unique and reproducible naming throughout the manuscript. Example: FibPLA instead of only PLA or ePLA if multiple PLA-based materials are used; ePLA is the name of a PLA from another manufacturer.
3D printer hardware	Report printer model, nozzle diameter/material and number of toolheads. Example: Original Prusa XL single-toolhead printer with 0.4 mm brass nozzle.
3D printing parameters	Report extrusion temperature, bed temperature, print speed, extrusion factor/flow rate, infill density and pattern, layer height, cooling settings, enclosure conditions, slicer software. Example: 220 °C nozzle temperature, 50 °C bed temperature, 100 % rectilinear infill, 0.2 mm layer height, no fan for first 5 layers, no enclosure, Prusa Slicer v.2.9.2.
Sample geometry	Describe sample dimensions, wall/perimeter settings, and orientation during printing and imaging. Example: cylindrical sample with specified diameter and height, one perimeter shell, upright print orientation.
CT imaging conditions	Report scanner model, acquisition protocol, tube voltage/current, reconstruction kernel, slice thickness, phantom environment, and sample positioning. Example: Siemens SOMATOM scanner, 120 kVp, 500 mA, Br40, sample positioned centrally in a PMMA phantom at CT isocentre.
Radiological measurements	Specify which quantities were measured (e.g. CT number, RED, RSP), the analysis method (SECT, DECT, peak finder, ion beam measurement), analysis software (3D slicer for HU analysis) and associated uncertainties.
Reproducibility and Stability	Assess repeatability across multiple prints and, if possible, across different filament batches. Example questions: How large is the variation between three identical samples? Does another batch of the same filament change CT number or RSP? Do equivalent materials from different vendors behave similarly? Does the material remain stable under irradiation or UV light?
Multi-material printing	Evaluate interfaces between materials and possible artefacts. Example questions: Are air gaps visible between materials? Are they detectable in CT images? Can toolhead calibration reduce interface gaps? Does warping or bed detachment occur? Are the selected materials thermally and mechanically compatible for co-printing?
Application-specific validation	Validate the printed object under the intended clinical or experimental conditions. Example questions: Does the measured RSP match the treatment planning prediction? Are deviations clinically relevant for the intended application?
